# Neuropsychological outcomes comparing deep brain stimulation and best medical treatment for Parkinson’s disease: a systematic review and meta-analysis

**DOI:** 10.3389/fpsyg.2026.1865587

**Published:** 2026-06-18

**Authors:** Jing Ning, Xiujuan Lv, Jianyu You, Xingyu Zhou, Chengfei An, Zhigang Zhou

**Affiliations:** 1 College of Clinical Medicine, Jiangxi University of Chinese Medicine, Nanchang, China; 2 Department of Rehabilitation Medicine, Anqiu City People's Hospital, Weifang, China; 3 School of Acupuncture-Moxibustion and Tuina, Jiangxi University of Chinese Medicine, Nanchang, China; 4 Acupuncture and Brain Science Research Center, Jiangxi University of Chinese Medicine, Nanchang, China

**Keywords:** cognitive function, deep brain stimulation, meta-analysis, neuropsychiatric symptoms, neuropsychological outcomes, Parkinson’s disease

## Abstract

**Background:**

Deep brain stimulation (DBS) is an established therapy for advanced Parkinson’s disease (PD). However, its impact on neuropsychological outcomes remains controversial and lacks systematic integration. This study aims to systematically evaluate the effects of DBS on cognitive and neuropsychiatric symptoms in PD patients through a meta-analysis of randomized controlled trials (RCTs).

**Methods:**

A systematic search was conducted in databases including PubMed, Embase, the Cochrane Library, and Web of Science for RCTs comparing DBS with best medical treatment (BMT). Cognitive outcome measures included Verbal Fluency test, the Stroop Color and Word Test, the Wisconsin Card Sorting Test (WCST), and the Mattis Dementia Rating Scale (MDRS). Neuropsychiatric outcome measures encompassed Part I of the Unified Parkinson’s Disease Rating Scale (UPDRS-I), the Beck Depression Inventory-II (BDI-II), the Montgomery-Asberg Depression Rating Scale (MADRS), as well as the Brief Anxiety Scale (BAS) and Beck Anxiety Inventory (BAI). Risk of bias was assessed using the Cochrane RoB 2 tool, and effect sizes were pooled using RevMan 5.4 software.

**Results:**

A total of 10 randomized controlled trials involving 1,274 patients were included. Meta-analysis results indicated that, compared to BMT, patients undergoing DBS showed a more significant decline in performance on Verbal Fluency test and the Stroop Color-Word Interference Test. No statistically significant differences were observed between the two groups regarding overall cognitive function, cognitive flexibility, depressive symptoms, anxiety symptoms, or overall neuropsychiatric symptoms. Sensitivity analysis suggested that the effect of DBS on anxiety symptoms may vary across studies, indicating a potential beneficial role of DBS in this domain.

**Conclusion:**

DBS treatment did not lead to widespread cognitive or neuropsychiatric decline in patients with PD based on group-level analyses. However, within the assessed cognitive domains, mild, specific negative effects were observed on higher-order executive functions. Clinical decision-making should incorporate individualized neuropsychological assessment and monitoring to balance the benefits of motor symptom improvement with potential cognitive risks.

**Systematic review registration:**

https://www.crd.york.ac.uk/PROSPERO/recorddashboard, this meta-analysis has been registered on the PROSPERO platform (Registration number: CRD420251273115).

## 1 Introduction

Parkinson’s disease (PD) is the second most common neurodegenerative disorder worldwide. According to the 2019 Global Burden of Disease Study (GBD 2016 Neurology Collaborators, 2019), PD affects approximately 8.5 million individuals globally, and its disease burden continues to rise. In addition to classic motor symptoms, non-motor symptoms represent a major source of disability in PD. Progressive impairment in Instrumental Activities of Daily Living (IADLs) is a key factor affecting the long-term independent living ability of PD patients, and such impairment is closely associated with neuropsychological symptoms (Giannouli and Tsolaki, 2019). Longitudinal studies indicate that about 40% of patients exhibit mild to moderate cognitive impairment at the time of diagnosis (Baiano et al., 2020), and a substantial proportion may progress to dementia as the disease advances. Neuropsychiatric symptoms such as depression and anxiety are also highly prevalent in PD patients (Sagna et al., 2014), with some manifestations appearing even several years before motor symptoms emerge (Schrag et al., 2015). These neuropsychological symptoms are key factors contributing to decline in the ability to live independently, increased caregiver burden, and rising healthcare costs, making them a critical focus in the comprehensive management of PD.

For patients with mid-to-late stage PD who respond poorly to medication or experience severe motor complications, deep brain stimulation (DBS) is an established and effective treatment. DBS involves implanting electrodes into specific brain targets, which are connected to a subcutaneous pulse generator, allowing for individualized parameter adjustment. Through electrical impulses, DBS modulates neural activity in target brain regions, thereby correcting abnormal neural signaling pathways underlying PD symptoms. Previous studies have confirmed that DBS significantly improves motor symptoms, enhances activities of daily living, and reduces levodopa requirements (Zhang et al., 2021). However, evidence regarding the impact of DBS on neuropsychological outcomes remains controversial and lacks systematic synthesis. Some studies suggest that DBS may have mild adverse effects on specific cognitive domains (Sisodia et al., 2024; Troster, 2024), while others report potential benefits in cognition, mood, and quality of life (Cartmill et al., 2021; Imbalzano et al., 2025). Although studies have systematically compared the effects of different stimulation targets on neuropsychological outcomes in PD patients (Elgebaly et al., 2018), a quantitative synthesis restricted exclusively to RCTs comparing DBS with best medical treatment remains lacking. Bucur et al. provided a comprehensive meta-analysis including both RCTs and observational studies (Bucur and Papagno, 2023), but an analysis limited to high-quality RCTs is still needed. Existing evidence is fragmented, heterogeneous, and inconsistent in reporting core outcome measures, making it difficult to establish clear consensus or define the overall efficacy of DBS in non-motor domains, thereby posing challenges for clinical decision-making.

Therefore, this study aims to systematically evaluate the impact of DBS on neuropsychological outcomes in PD patients by conducting a systematic review and meta-analysis of all available RCT evidence, with the goal of providing more direct and reliable evidence to inform clinical practice.

## 2 Methods

### 2.1 Protocol and registration

This meta-analysis has been registered on the PROSPERO platform (Registration number: CRD420251273115). The study protocol was developed in accordance with the Preferred Reporting Items for Systematic Reviews and Meta-Analyses (PRISMA) guidelines.

### 2.2 Data sources and searches

This meta-analysis systematically searched for randomized controlled trials (RCTs) concerning deep brain stimulation (DBS) for the treatment of Parkinson’s disease (PD) in the Cochrane Library, PubMed, Embase, and Web of Science databases. The search timeframe was from the inception of each database to December 24, 2025. Keywords included “Parkinson Disease,” “Deep Brain Stimulation”, “Non-motor Symptoms”, “Cognition”, “Executive Function”, “Depression”, “Anxiety”, “Verbal Fluency”, “Dementia”, “Memory”, among others, with the search limited to RCTs. A computerized search was conducted using a combination of subject headings and free-text terms. The detailed search strategy is available in Supplementary material.

### 2.3 Inclusion and exclusion criteria

Inclusion criteria: (1) Study design: RCTs with a follow-up period of no less than 3 months. (2) Study participants: Patients diagnosed with idiopathic Parkinson’s disease. (3) Interventions: The control group received best medical treatment (BMT); the experimental group underwent DBS, targeting any nucleus, either in addition to BMT or as a standalone therapy. (4) Outcome measures: Cognitive function was assessed using the Verbal Fluency test, the Stroop Color and Word Test (which consists of three conditions: Word Reading, Color Naming, and Color-Word Interference), the Wisconsin Card Sorting Test (WCST), and the Mattis Dementia Rating Scale (MDRS). Overall mental status was assessed using Part I of the Unified Parkinson’s Disease Rating Scale (UPDRS-I). Depressive symptoms were assessed using the Beck Depression Inventory-II (BDI-II) and the Montgomery-Åsberg Depression Rating Scale (MADRS). Anxiety symptoms were assessed using the Brief Anxiety Scale (BAS) and the Beck Anxiety Inventory (BAI). Studies employing one or more of the above scales were eligible for inclusion.

Exclusion criteria: (1) Non-RCTs. (2) Full text unavailable or duplicate publications. (3) Reviews, animal studies, letters, conference abstracts, and dissertations. (4) Studies with major flaws in design, missing data, or unclear outcome measures. (5) Non-English literature.

The selection of outcome measures was guided by the following considerations: (1) To ensure consistency with established consensus recommendations for neuropsychological assessment in PD, we prioritized tests that have been validated in large-scale PD cohorts and are commonly reported across RCTs. (2) To enable quantitative synthesis, we only included outcomes reported by at least three independent studies. This criterion excluded domains such as memory and visuospatial function due to insufficient reporting consistency across eligible RCTs. (3) Regarding neuropsychiatric symptoms, we acknowledge that UPDRS-I is not a dedicated depression or anxiety scale; however, it was included as a measure of overall neuropsychiatric burden given its wide use in DBS RCTs, while depression and anxiety were analyzed separately using validated scales.

### 2.4 Study selection and data extraction

Initially, duplicate records were removed using EndNote X9 reference management software. Subsequently, two researchers independently screened the remaining records. During the screening process, articles that were clearly irrelevant were first excluded based on their titles and abstracts. The full texts of the remaining articles were then obtained and rigorously assessed for eligibility according to the predefined inclusion and exclusion criteria. Finally, two researchers independently extracted data and cross-checked their results. Any disagreements were resolved through discussion, with the involvement of a third researcher if necessary, until a consensus was reached. Extracted information included: basic study characteristics (e.g., first author, publication year), sample size, patient age and disease duration, intervention details, and outcome measures.

If multiple publications originated from the same clinical trial, the following principles were followed to ensure each independent sample contributed data only once per meta-analysis: for studies reporting identical outcomes, the publication with the longest follow-up and most complete data reporting was selected. For studies reporting different outcomes, they were treated as independent data sources, and their data were included in the respective meta-analyses based on the reported outcomes. During data extraction, it was ensured that all data used to calculate a single effect size were sourced from the same report.

### 2.5 Assessment of study quality

Two researchers independently assessed the risk of bias using the Cochrane Risk of Bias 2 (RoB 2) tool (Sterne et al., 2019). Their assessments were cross-checked, and any discrepancies were resolved through consensus discussion or, if required, by consulting a third party. The evaluation covers the five core domains of the RoB 2 tool: randomization process, deviations from intended interventions, missing outcome data, bias in measurement of the outcome, and selective reporting of results.

### 2.6 Data synthesis and analysis

This meta-analysis was performed using RevMan 5.4. For continuous outcomes, the synthesis of effect sizes prioritized the use of change-from-baseline values. Continuous variables with consistent units were expressed as mean differences (MD) with 95% confidence intervals (CIs); for those with inconsistent units, standardized mean differences (SMD) were used. Dichotomous variables were presented as risk ratios (RR) with 95% CIs. For multi-arm trials, in accordance with the Cochrane Handbook for Systematic Reviews of Interventions, all relevant intervention groups were pooled into a single intervention group, and all relevant control groups were pooled into a single control group for analysis, so as to maintain the independence of individual studies and avoid double-counting. Heterogeneity was assessed using the *p*-value and I^2^ statistic. In the absence of significant heterogeneity (I^2^ < 50%, *p* > 0.1), a fixed-effects model was applied. If significant heterogeneity existed (I^2^ > 50%, *p* < 0.1), sensitivity analysis was conducted by sequentially excluding individual studies to evaluate the robustness of the results, followed by subgroup analysis to explore potential sources of heterogeneity. If heterogeneity remained unresolved, a random-effects model was employed for pooled analysis. A p-value < 0.05 was considered statistically significant. Publication bias was assessed via funnel plot symmetry if more than 10 studies were included for a specific outcome.

### 2.7 GRADE evidence level evaluation

Two researchers independently evaluated the quality of evidence for each outcome using the GRADE framework (Guyatt et al., 2008). Five domains were evaluated to identify factors that might lead to downgrading the evidence quality: risk of bias, inconsistency, indirectness, imprecision, and publication bias. The quality of evidence was categorized into four grades: high, moderate, low, and very low.

## 3 Results

### 3.1 Study selection and characteristics

**Figure 1:**
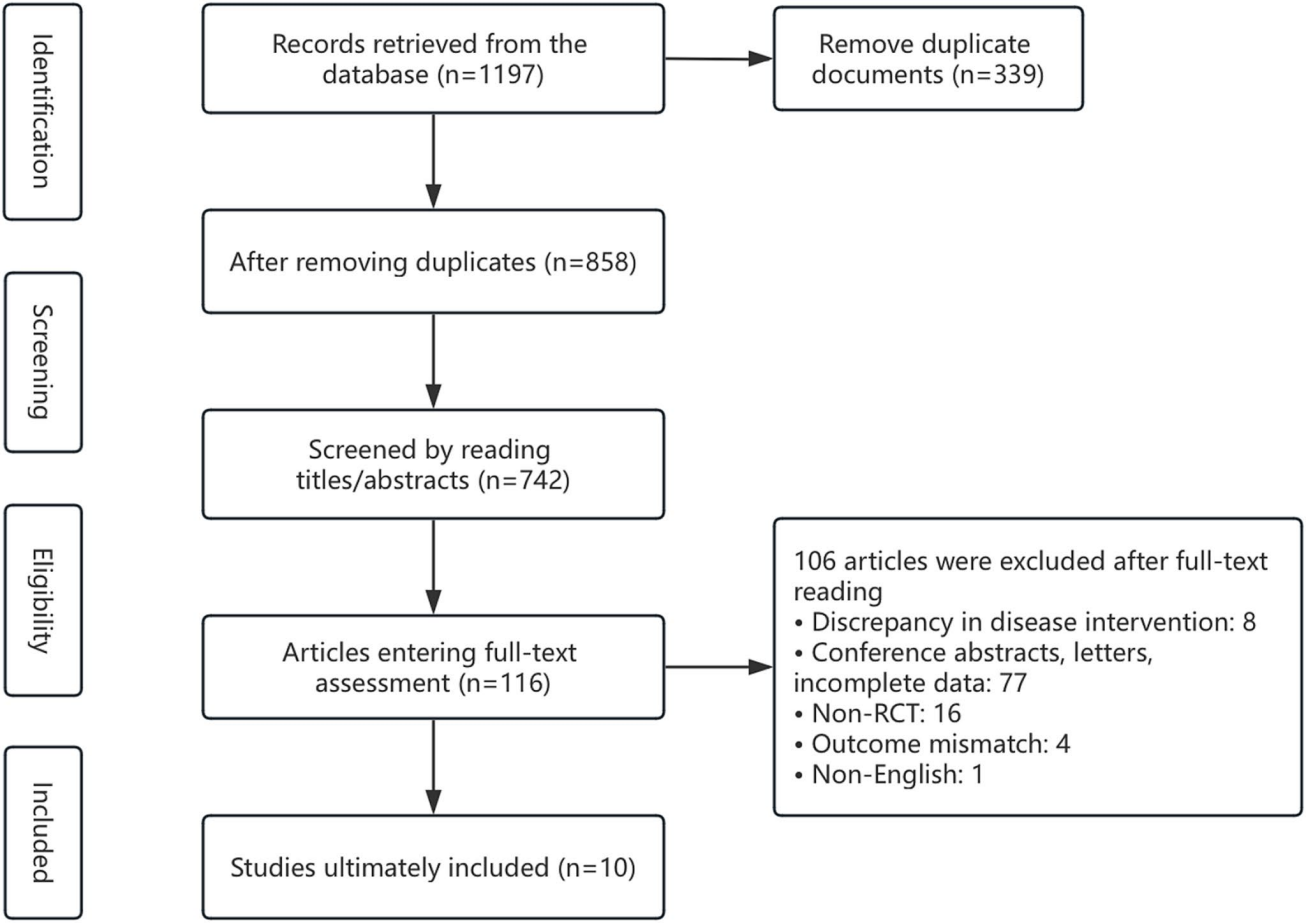
Literature selection process.

The initial search identified 1,197 records. After removing 339 duplicates, 858 records underwent initial screening. Based on title and abstract assessment, 742 records were excluded. The full texts of the remaining 116 articles were reviewed, resulting in the exclusion of 106 articles. Ultimately, 10 articles were included in the meta-analysis (Deuschl et al., 2006; Schuepbach et al., 2007; Witt et al., 2008; Williams et al., 2010; Schuepbach et al., 2013; Rothlind et al., 2015; Tramontana et al., 2015; Philipson et al., 2021; Hacker et al., 2023a; Hacker et al., 2023b), among which two articles were derived from the same clinical trial (Hacker et al., 2023a; Hacker et al., 2023b). The study selection process is detailed in Figure 1.

The 10 included studies enrolled a total of 1,274 patients. The DBS surgical targets included the subthalamic nucleus (STN), the globus pallidus internus (GPi), and the caudal zona incerta (cZi). The basic characteristics of the included studies are presented in Table 1.

**Table 1:** Characteristics of studies included in the analysis.

References	Recruited sample size	Average age (y)		PD duration (y)		Intervention		Follow-up duration (y)	Outcomes
		T	C	T	C	T	C		
Deuschl et al. (2006)	156	60.5 ± 7.4	60.8 ± 7.8	13.0 ± 5.8	13.8 ± 5.6	STN-DBS + BMT	BMT	0.5y	4,7
Schuepbach et al. (2007)	20	48.5 ± 3.0	6.8 ± 1.0	STN-DBS	BMT	1.5	4,7,9
Witt et al. (2008)	123	60.2 ± 7.9	59.4 ± 7.5	13.8 ± 6.3	14.0 ± 6.1	STN-DBS	BMT	0.5y	1,2,4,5,6,8
Williams et al. (2010)	366	59	11.5	11.2	STN-DBS + BMT	BMT	1y	4,5
Schuepbach et al. (2013)	251	52.6	7.5	STN-DBS + BMT	BMT	2y	4,5,6
Rothlind et al. (2015)	280	61.7 ± 8.8	11.0	12.8	GPi/STN-DBS+BMT	BMT	0.5y	1,2,3
Tramontana et al. (2015)	30	60 ± 6.8	60 ± 7.0	2.2 ± 1.4	2.1 ± 1.1	STN-DBS + BMT	BMT	2y	1,2,3
Philipson et al. (2021)	18	55.6 ± 11.3	60.3 ± 8.0	6.4 ± 3.0	10.3 ± 5.6	cZi-DBS + BMT	BMT	0.5y	3,6
Hacker et al. (2023a)	30	61.4 ± 6.4	60.7 ± 6.6	2.2 ± 1.4	2.0 ± 1.0	STN-DBS + BMT	BMT	11y	5
Hacker et al. (2023b)	5y	1,2,3

DBS, deep brain stimulation; STN, subthalamic nucleus; GPi, globus pallidus internus; cZi, caudal zona incerta; BMT, best medical treatment; 1, Verbal Fluency test;2, Stroop Color and Word Test;3, Wisconsin Card Sorting Test;4, Mattis Dementia Rating Scale;5, Part I of the Unified Parkinson’s Disease Rating Scale;6, Beck Depression Inventory-II;7, Montgomery-Asberg Depression Rating Scale;8, Beck Anxiety Inventory;9, Brief Anxiety Scale.

### 3.2 Risk of bias assessment results

**Figure 2:**
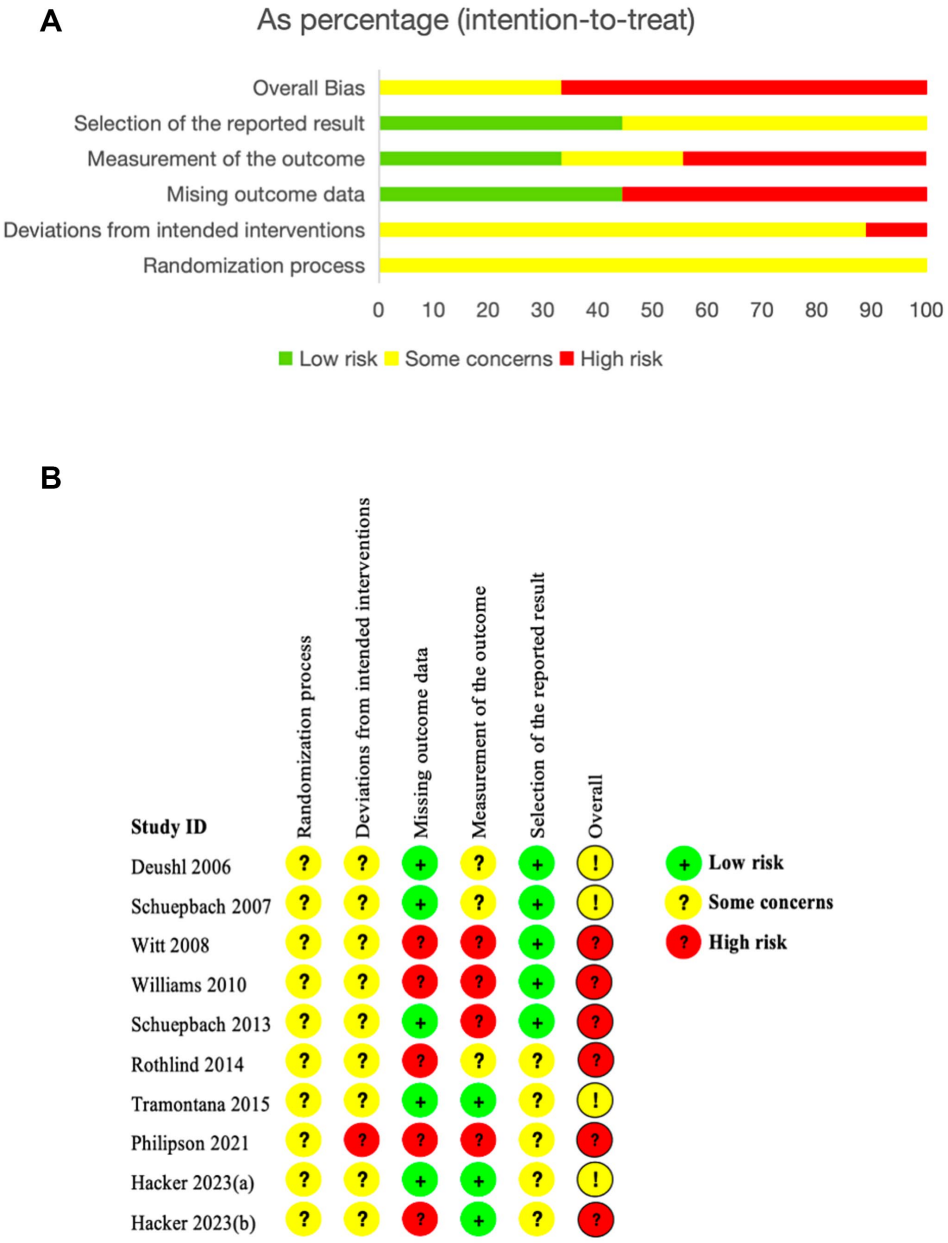
Risk of bias assessment of included randomized controlled trials using the Cochrane Risk of Bias 2 (RoB 2) tool. **(A)** The graph of risk of bias summary showing the percentage distribution of bias judgments across each RoB 2 domain; **(B)** Risk of bias assessment across domains presenting study-specific domain ratings. Green circles indicate low risk of bias, yellow symbols/question marks indicate some concerns, and red circles indicate high risk of bias.

The methodological quality of all included studies was assessed using the ROB 2.0 tool. Most studies reported randomization procedures; however, due to insufficient description of allocation concealment, they were rated as having some concerns in the domain of randomization process. In the domain of deviations from intended interventions, the majority of studies were judged as having some concerns because they did not explicitly apply intention-to-treat analysis, and both participants and intervention providers were aware of group assignments. One study was rated as high risk in this domain due to the occurrence of surgery-related serious adverse events and imbalanced deviations between groups, which could substantially affect neuropsychological outcomes. Regarding missing outcome data, five studies were rated as high risk owing to significant data attrition or high dropout rates. In the measurement of outcomes, most studies were rated as high risk or having some concerns because outcome assessors were not adequately blinded, or inconsistencies were present in the measurement tools or methods applied. In the selection of reported results, five studies were judged as having some concerns, primarily because it was unclear whether a prespecified statistical analysis plan had been finalized prior to unblinding. Overall, six studies were rated as high risk in at least one key domain, and four studies were rated as having some concerns. The methodological quality of the body of evidence is associated with a high risk of bias, which warrants cautious interpretation of the findings. The risk of bias summary graph and assessment table are presented in Figure 2.

### 3.3 Meta-analysis results

#### 3.3.1 Effect of DBS on cognitive function in PD patients

**Figure 3:**
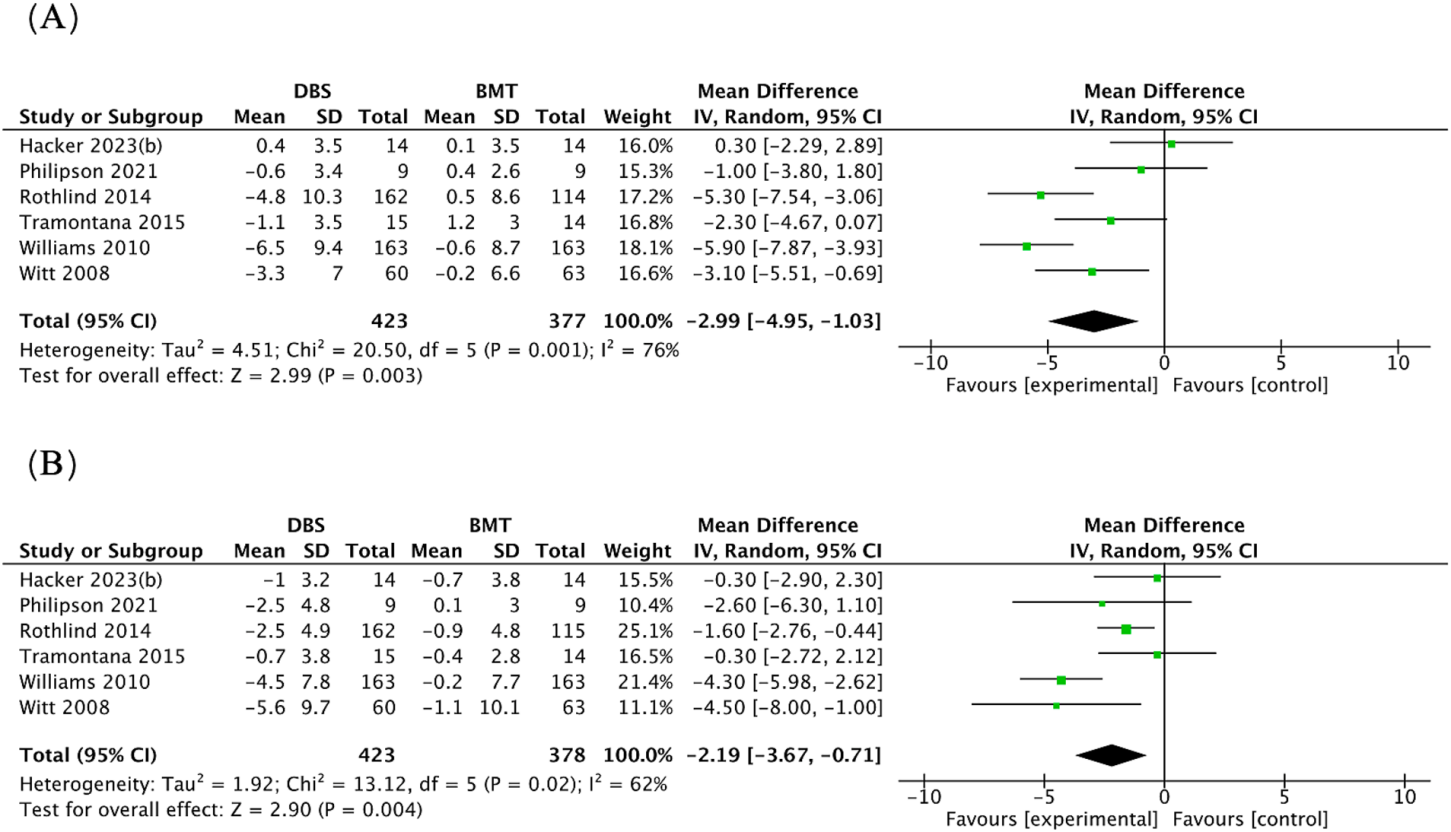
Forest plot of Verbal Fluency in DBS versus BMT groups for patients with PD. **(A)** Phonemic verbal fluency; **(B)** Semantic verbal fluency. Study-specific mean differences (MDs) are shown as squares, with areas proportional to study weights. Horizontal bars depict 95% confidence intervals (CIs), and the pooled estimate is displayed as a diamond derived from a random-effects model. The vertical reference line at 0 indicates no overall effect.

Verbal fluency tests, typically comprising phonemic and semantic fluency tasks, are commonly used to assess executive function, language ability, and cognitive flexibility, serving as standard tools in neuropsychological assessment. Six studies included both phonemic and semantic fluency tests. The analysis for phonemic verbal fluency included 800 patients. Due to significant heterogeneity (*p* = 0.001, I^2^ = 76%), a sensitivity analysis was performed by sequentially removing each study; the influence of individual studies on the overall result was minimal, indicating robust findings. Using a random-effects model, the pooled effect size showed a statistically significant difference between DBS and BMT on phonemic fluency in PD patients (MD = −2.99, 95% CI -4.95 to −1.03, *p* = 0.003). The analysis for semantic verbal fluency included 801 patients, also with considerable heterogeneity (*p* = 0.02, I^2^ = 62%). Sensitivity analysis confirmed result robustness. The random-effects model revealed a statistically significant difference between DBS and BMT on semantic fluency (MD = −2.19, 95% CI -3.67 to −0.71, *p* = 0.004). These findings show that, compared to patients receiving BMT, those treated with DBS experienced a statistically significant decline in both phonemic and semantic verbal fluency over a follow-up period of 6 months to 5 years. Details are shown in Figure 3.

**Figure 4:**
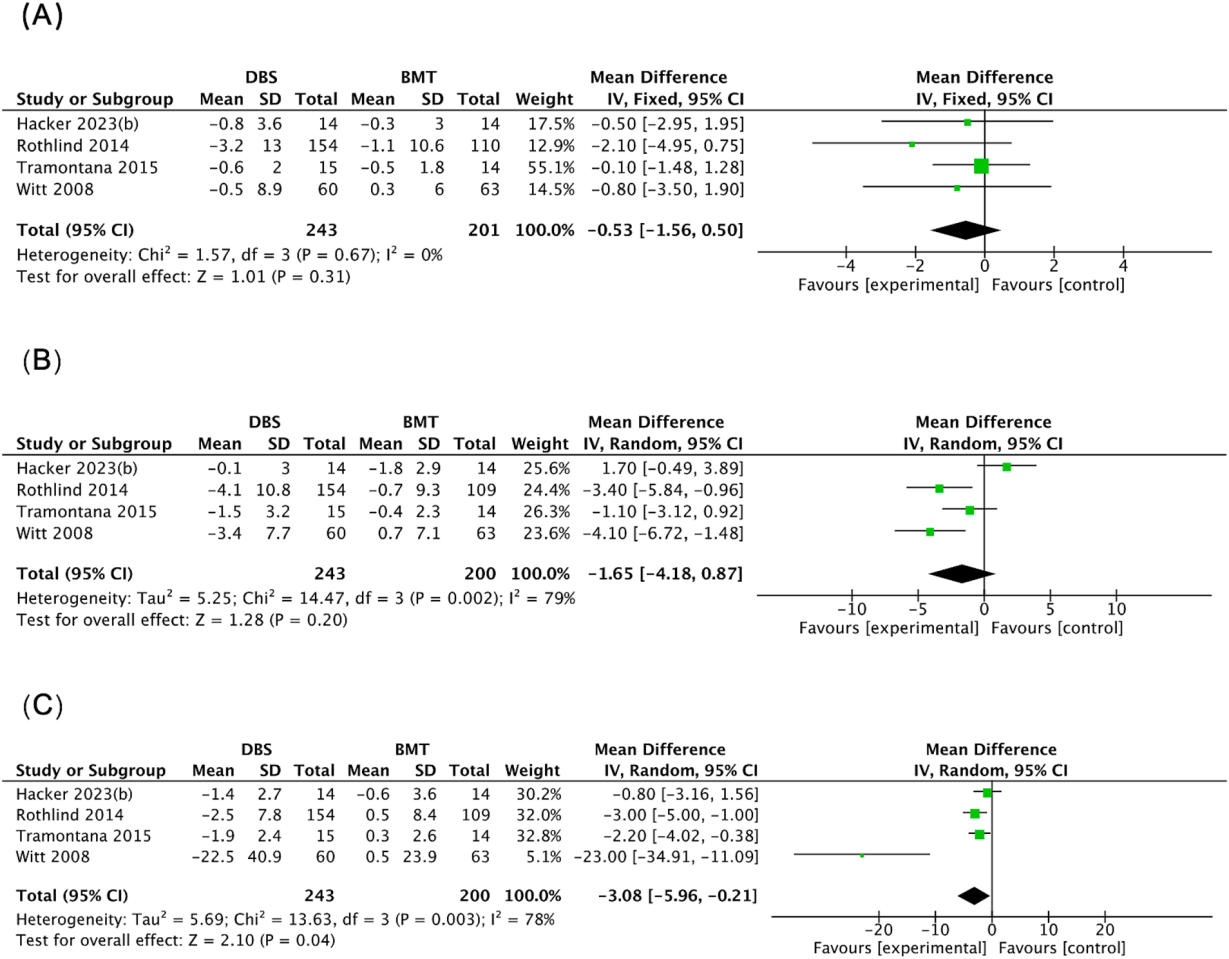
Forest plot of the Stroop Color and Word Test in DBS versus BMT groups for patients with PD. **(A)** Stroop Word Reading; **(B)** Stroop Color Naming; **(C)** Stroop Color-Word Interference. Study-specific mean differences (MDs) are shown as squares, with areas proportional to study weights. Horizontal bars represent 95% confidence intervals (CIs). The pooled estimates are displayed as diamonds: a fixed-effects model was used for subtest **(A)**, while random-effects models were applied for subtests **(B,C)**. The vertical reference line at 0 indicates no overall effect.

The Stroop Color and Word Test is a classic tool for revealing deficits in inhibitory control and prefrontal executive dysfunction in patients with Parkinson’s disease (PD). Four studies administered this test to PD patients. Stroop Word Reading was assessed in 444 patients. Forest plot analysis indicated no heterogeneity (*p* = 0.67, I^2^ = 0%), and a fixed-effects model was applied. The pooled effect size showed no statistically significant difference between DBS and BMT on Stroop Word Reading performance in PD patients (MD = −0.53, 95% CI − 1.56 to 0.50, *p* = 0.31). Stroop Color Naming was assessed in 443 patients. Significant heterogeneity was observed (*p* = 0.002, I^2^ = 79%). A leave-one-out sensitivity analysis was performed, and the results remained robust. Using a random-effects model, the pooled effect size indicated no statistically significant difference between DBS and BMT on Stroop Color Naming (MD = −1.65, 95% CI -4.18 to 0.87, *p* = 0.20). Stroop Color-Word Interference was assessed in 443 patients. Significant heterogeneity was present (*p* = 0.003, I^2^ = 78%). Sensitivity analysis confirmed the robustness of the findings. A random-effects model revealed a statistically significant difference between DBS and BMT on Stroop Color-Word performance (MD = −3.08, 95% CI -5.96 to −0.21, *p* = 0.04). These findings show that, compared to patients receiving BMT, those treated with DBS experienced a statistically significant decline only in the Stroop Color-Word Interference test, but not in the Word Reading or Color Naming tests. Details are presented in Figure 4.

**Figure 5:**
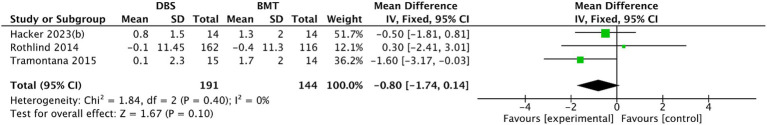
Forest plot of the Wisconsin Card Sorting Test (WCST) in DBS versus BMT groups for patients with PD. Study-specific mean differences (MDs) are shown as squares, with areas proportional to study weights. Horizontal bars depict 95% confidence intervals (CIs), and the pooled estimate is displayed as a diamond derived from a fixed-effects model. The vertical reference line at 0 indicates no overall effect.

WCST is widely used to assess cognitive flexibility and abstract problem-solving ability and is a classical task in neuropsychology. Three studies reported data from the Perseverative Errors component of the WCST, involving 335 patients. Forest plot analysis showed no heterogeneity (*p* = 0.40, I^2^ = 0%), and a fixed-effects model was applied. The pooled effect size revealed no statistically significant difference between DBS and BMT on WCST Perseverative Errors in PD patients (MD = −0.80, 95% CI − 1.74 to 0.14, *p* = 0.10). These findings show that, within the follow-up period of the included studies, DBS therapy did not produce a statistically significant effect on cognitive flexibility or abstract problem-solving ability in PD patients compared to best medical treatment. See Figure 5 for details.

**Figure 6:**
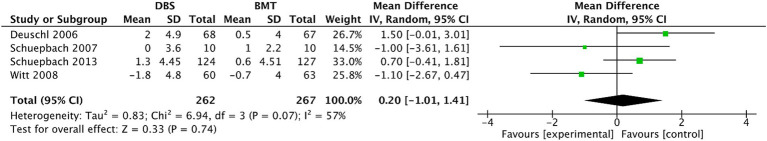
Forest plot of the Mattis Dementia Rating Scale in DBS versus BMT groups for patients with PD. Study-specific mean differences (MDs) are shown as squares, with areas proportional to study weights. Horizontal bars depict 95% confidence intervals (CIs), and the pooled estimate is displayed as a diamond derived from a random-effects model. The vertical reference line at 0 indicates no overall effect.

MDRS is commonly used to evaluate overall cognitive function and is a standard tool for clinical dementia screening. Four studies administered the MDRS, involving 529 patients. Considerable heterogeneity was observed (*p* = 0.07, I^2^ = 57%), and a leave-one-out sensitivity analysis was conducted. The influence of individual studies on the overall result was minimal, confirming the robustness of the findings. Using a random-effects model, the pooled effect size showed no statistically significant difference between DBS and BMT on MDRS scores in PD patients (MD = 0.20, 95% CI − 1.01 to 1.41, *p* = 0.74). These findings show that, within the available follow-up period, DBS therapy did not significantly affect overall cognitive function in PD patients. See Figure 6 for details.

#### 3.3.2 Effect of DBS on neuropsychiatric symptoms in PD patients

**Figure 7:**
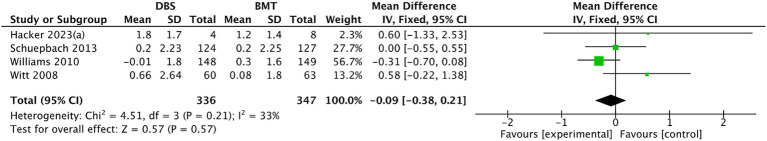
Forest plot of UPDRS-I scores in DBS versus BMT groups for patients with PD. Study-specific mean differences (MDs) are shown as squares, with areas proportional to study weights. Horizontal bars depict 95% confidence intervals (CIs), and the pooled estimate is displayed as a diamond derived from a fixed-effects model. The vertical reference line at 0 indicates no overall effect.

UPDRS-I uses a semi-structured clinical interview to quantify common mental, behavioral, and mood disturbances in PD patients, and is one of the gold-standard instruments for evaluating the progression of non-motor symptoms. Four studies assessed patients using the UPDRS-I, involving 683 patients. Forest plot analysis indicated low heterogeneity (*p* = 0.21, I^2^ = 33%), and a fixed-effects model was applied. The pooled effect size showed no statistically significant difference between DBS and BMT on UPDRS-I scores in PD patients (MD = −0.09, 95% CI –0.38 to 0.21, *p* = 0.57). This indicates that, compared with BMT, DBS therapy did not exert a statistically significant effect on neuropsychiatric symptoms in PD patients. See Figure 7 for details.

**Figure 8:**
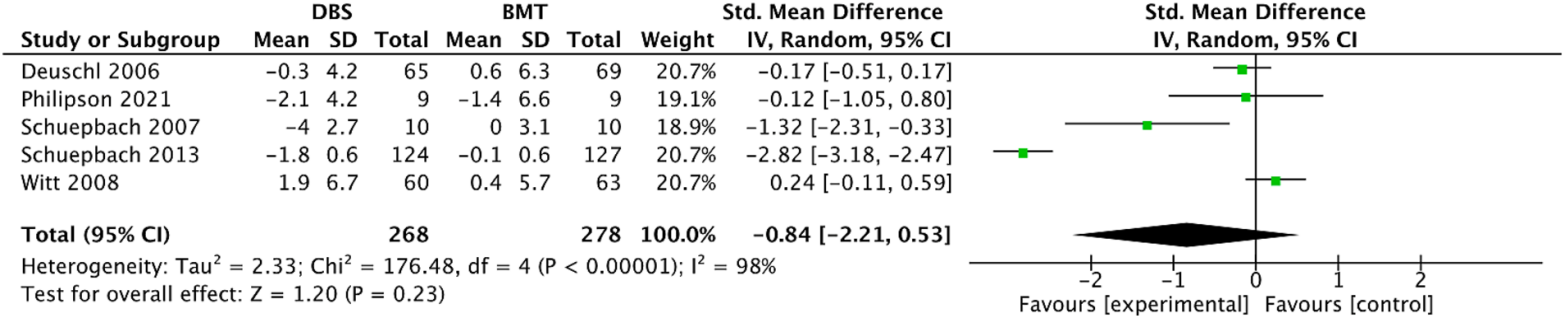
Forest plot of depressive symptoms in DBS versus BMT groups for patients with PD. Study-specific standardized mean differences (SMDs) are shown as squares, with areas proportional to study weights. Horizontal bars depict 95% confidence intervals (CIs), and the pooled estimate is displayed as a diamond derived from a random-effects model. The vertical reference line at 0 indicates no overall effect.

Five studies evaluated depressive symptoms using either the BDI-II or the MADRS, involving 546 patients. Substantial heterogeneity was observed (*p* < 0.00001, I^2^ = 98%). A leave-one-out sensitivity analysis was performed, and the influence of individual studies on the overall result was minimal, confirming the robustness of the findings. Using a random-effects model, the pooled effect size revealed no statistically significant difference between DBS and BMT on depression ratings in PD patients (SMD = −0.84, 95% CI –2.21 to 0.53, *p* = 0.23). This suggests that, based on the available evidence, DBS therapy did not demonstrate a statistically significant advantage over BMT in improving depressive symptoms in PD patients. See Figure 8 for details.

**Figure 9:**
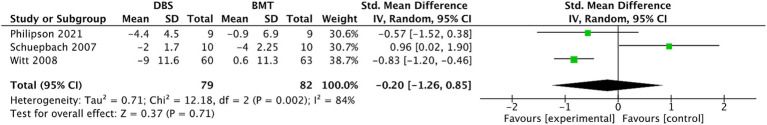
Forest plot of anxiety symptoms in DBS versus BMT groups for patients with PD. Study-specific standardized mean differences (SMDs) are shown as squares, with areas proportional to study weights. Horizontal bars depict 95% confidence intervals (CIs), and the pooled estimate is displayed as a diamond derived from a random-effects model. The vertical reference line at 0 indicates no overall effect.

Three studies used the BAI or BAS to assess anxiety in patients, involving a total of 161 individuals. Forest plot analysis revealed substantial heterogeneity (*p* = 0.002, I^2^ = 84%), and a random-effects model was applied. The pooled effect size indicated no statistically significant difference between DBS and BMT on anxiety scores in PD patients (SMD = −0.20, 95% CI –1.26 to 0.85, *p* = 0.71). This suggests that, based on current evidence, DBS therapy does not show a statistically significant difference from BMT in improving anxiety symptoms in PD patients. A sensitivity analysis performed by sequentially excluding each study found that after removing the study by Schuepbach et al., the direction of the pooled effect changed and reached statistical significance (SMD = −0.80, 95% CI –1.14 to −0.46, *p* < 0.00001), indicating a potential advantage of DBS in alleviating anxiety. These findings imply that the evidence regarding the effect of DBS on anxiety symptoms in PD patients remains inconsistent and conclusions are not robust. Future studies with larger sample sizes, more uniform assessment tools, and more homogeneous patient baselines are needed to validate these results. See Figure 9 for details.

### 3.4 Quality of evidence

The GRADE assessment indicated that, due to the combined impact of risk of bias and heterogeneity among the included studies, the overall certainty of evidence was rated as low for most outcome measures, including the Verbal Fluency test, Stroop Color Naming, Stroop Color-Word performance, and MDRS. Furthermore, the certainty of evidence for outcomes related to depressive symptoms and anxiety symptoms was downgraded to very low, owing to the additional effects of risk of bias, very substantial inconsistency, and indirectness arising from the use of differing measurement instruments. By contrast, the certainty of evidence for Stroop word reading, WCST, and the UPDRS-I was rated as moderate, having been downgraded once primarily because of risk of bias. A detailed summary of the ratings and reasons for downgrading is provided in Table 2.

**Table 2:** GRADE evidence profile for primary outcomes.

Outcomes		Risk of bias	Inconsistency	Indirectness	Imprecision	Publication bias	Certainty
Verbal fluency	Phonemic verbal fluency	−1	−1	0	0	0	⊕ ⊕ ⊝⊝ Low
	Semantic verbal fluency	−1	−1	0	0	0	⊕ ⊕ ⊝⊝ Low
Stroop color and word test	Word reading	−1	0	0	0	0	⊕ ⊕ ⊝⊝ Moderate
	Color naming	−1	−1	0	0	0	⊕ ⊕ ⊝⊝ Low
	Color-word interference	−1	−1	0	0	0	⊕ ⊕ ⊝⊝ Low
Other cognitive functions	WCST	-1	0	0	0	0	⊕ ⊕ ⊝⊝ Moderate
	MDRS	-1	-1	0	0	0	⊕ ⊕ ⊝⊝ Low
Neuropsy- chiatric symptoms	Depressive symptoms	-1	−2	−1	0	0	⊕ ⊕ ⊝⊝ Very low
	Anxiety symptoms	−1	−2	−1	−1	0	⊕ ⊕ ⊝⊝ Very low
	UPDRS-I	−1	0	0	0	0	⊕ ⊕ ⊝⊝ Moderate

Risk of bias: −1, DBS studies cannot be fully blinded, combined with other potential biases, leading to an overall judgment of serious risk of bias. Inconsistency: -1, I^2^ > 50%; −2, I^2^ > 75%. Indirectness: −1, Pooled analysis of depressive and anxiety symptoms using different assessment tools introduces indirect comparison. Imprecision: −1, The sample size for anxiety symptoms is too small, resulting in unstable findings. Publication bias: Each meta-analysis included fewer than 10 studies, formal assessment was not performed, and no obvious signs of publication bias were detected.

## 4 Discussion

### 4.1 Summary of evidence and mechanistic interpretation

This systematic review and meta-analysis comprehensively evaluated the impact of DBS on neuropsychological outcomes in patients with Parkinson’s disease (PD). The results indicate that the effect of DBS on cognitive function is markedly task-specific. Performance on verbal fluency tasks and the color-word interference condition of the Stroop test declined significantly in the DBS group compared to the BMT group. In contrast, no statistically significant differences were found between the two groups regarding overall cognitive function, cognitive flexibility, or neuropsychiatric symptoms. These findings suggest that while DBS provides substantial improvement in motor symptoms of PD, its impact on most neuropsychological domains is relatively neutral, with minor negative effects observed specifically in higher-order cognitive functions.

Specifically, DBS was associated with declines in both phonemic and semantic verbal fluency, suggesting a selective effect on language production and retrieval processes. In the Stroop test, performance declined only under the color-word interference condition, which has a high demand for inhibitory control, whereas the simpler word reading and color naming tasks showed no decline. Global cognitive function (MDRS) and cognitive flexibility (WCST) also did not significantly decrease. This pattern of effects indicates that the cognitive impact of DBS is not global, but rather selectively affects cognitive tasks that rely on language production/retrieval and complex executive control. Previous studies have confirmed that the completion of such tasks is highly dependent on the functional integrity of the frontal-striatal circuits (Frank et al., 2007; Jahanshahi et al., 2015). Accordingly, the selective cognitive effects observed in this study may reflect the vulnerability of the frontal-striatal circuits to DBS neuromodulation, while the more distributed networks supporting global cognition remain relatively preserved.

The task-specific cognitive effects of DBS in PD patients are likely related to its complex modulatory role on the neurodynamics of the basal ganglia-thalamocortical circuits. DBS is thought to modulate information transfer within prefrontal-striatal networks, potentially interfering with the timing of neural processing required for verbal retrieval and cognitive control (Frank et al., 2007; Jahanshahi et al., 2015). Functional neuroimaging studies have shown that DBS can alter activation patterns in prefrontal and cingulate cortices during tasks involving response selection and conflict monitoring (Schroeder et al., 2002; Ballanger et al., 2009), processes that are central to verbal fluency and Stroop performance. Electrophysiological evidence further supports this interpretation. High-frequency DBS may disrupt the oscillatory rhythms that facilitate efficient information transfer in prefrontal-striatal circuits, thereby affecting task performance (Lofredi et al., 2023). Verbal fluency tasks, for instance, depend on precisely timed neural transmission along the fronto-striatal pathway; disruption of oscillatory patterns may contribute to the observed decline. In contrast, we found no significant decline in global cognitive function or cognitive flexibility. This discrepancy may reflect the differential sensitivity of these measures: global cognition is supported by broader neural networks that may possess greater compensatory capacity, whereas tasks requiring rapid, integrative processing within specific frontal-striatal circuits are more vulnerable to DBS-related disruption. Regarding mood outcomes, our meta-analysis did not identify a significant overall effect of DBS on depression or anxiety. However, the substantial heterogeneity observed suggests that the impact on mood is not uniform. Variability in stimulation targets, patient characteristics, and assessment tools likely contributes to these inconsistent findings. Additionally, improvements in mood may arise indirectly from motor symptom relief rather than direct modulation of limbic circuits. Collectively, these findings underscore the complexity of DBS effects on mood and highlight the need for individualized assessment.

### 4.2 Comparison with previous meta-analyses

Previous meta-analyses have examined neuropsychological outcomes following DBS in PD (Elgebaly et al., 2018; Bucur and Papagno, 2023). Elgebaly et al. focused specifically on comparing STN versus GPi targets, concluding that target selection may influence specific cognitive outcomes. Bucur et al. conducted a comprehensive meta-analysis including both RCTs and observational studies with long-term follow-up, reporting that DBS is associated with declines in verbal fluency but not in global cognition.

The present meta-analysis differs from and extends these prior works in several ways. First, unlike Elgebaly et al., we did not compare targets but instead restricted inclusion to RCTs with a BMT control group, allowing us to estimate the net effect of DBS as a treatment modality relative to standard medical therapy. Second, whereas Bucur et al. included both RCTs and observational studies, we limited our analysis to RCTs only, thereby minimizing confounding by study design and providing higher-level evidence. Third, we applied the GRADE framework to assess evidence certainty, which has not been systematically reported in prior meta-analyses in this area.

### 4.3 Limitations and future perspectives

This study has several limitations. First, the number of included randomized controlled trials was limited, with the vast majority focusing on the STN target. There is a notable scarcity of studies investigating other targets. Moreover, substantial heterogeneity existed among the studies regarding stimulation parameters, baseline patient characteristics, and follow-up durations, which significantly constrained our ability to perform target-specific subgroup analyses. Existing evidence suggests that different DBS targets may differentially affect neuropsychological outcomes (Follett et al., 2010). Consequently, the results and conclusions of this meta-analysis are more applicable to STN-DBS, with limited generalizability to targets like the GPi and cZi. Second, the heterogeneity of outcome measures reported in the included randomized controlled trials was substantial, which limited our ability to assess other clinically relevant cognitive domains such as memory and visuospatial function. Apathy, a common neuropsychiatric symptom in patients with PD, is closely associated with executive dysfunction and can affect neuropsychological performance independently of depression (Giannouli and Tsolaki, 2021). None of the included trials provided validated standardized assessment data for apathy, which further constrained our analysis of this key confounding variable. Third, due to the nature of the surgical intervention, complete blinding of patients and assessors was challenging, potentially introducing performance and measurement biases. Fourth, the follow-up periods in most studies remain medium-to-short term for observing the long-term cognitive evolution in neurodegenerative diseases. The progressive neurodegenerative course of PD itself intertwines with the treatment effects of DBS over the long term, and their net effect requires clarification through longer-term follow-up data. Finally, this analysis was primarily based on group-average effects and did not examine the influence of individual differences—such as preoperative cognitive baseline, precision of electrode placement, and personalized stimulation parameter settings—on neuropsychological outcomes.

Based on these findings and limitations, future research should advance in the following directions: First, conducting well-designed, long-term follow-up randomized controlled trials that systematically compare the differential effects of various DBS targets on neuropsychological outcomes, combined with individualized electrode targeting and stimulation parameter optimization. Second, deeply integrating multimodal assessment approaches by combining detailed neuropsychological evaluations with task-based fMRI, resting-state EEG/MEG, and local field potential recordings to elucidate the real-time neural circuit mechanisms through which DBS affects specific cognitive functions. Third, striving to develop clinical prediction models that integrate patients’ preoperative clinical features, neuroimaging biomarkers, and genetic information to predict the individual neuropsychological risks and benefits, enabling truly personalized treatment decision-making. Fourth, at the level of clinical practice, comprehensive neuropsychological assessment should be established as a core component of both preoperative evaluation and long-term postoperative follow-up for DBS. The results of this study show that, compared with BMT, the DBS group exhibited decline only in verbal fluency and executive control tasks. Preoperative assessment should therefore focus on these vulnerable cognitive domains, and postoperative monitoring should repeat these assessments, especially during the first year after surgery, while maintaining routine follow-up of all other neuropsychological outcomes. Future trials could consider adopting standardized core outcome sets for neuropsychological assessment in PD-DBS; for example, including validated apathy scales would help evaluate the potential confounding role of apathy in postoperative cognitive changes. Meanwhile, for patients who show specific cognitive declines, combined intervention strategies incorporating cognitive rehabilitation training or non-invasive brain stimulation could be explored to optimize overall treatment outcomes. Thus, the benefit–risk balance of DBS should be evaluated on an individual basis, integrating motor, cognitive, and psychiatric profiles.

## 5 Conclusion

In summary, current evidence from randomized controlled trials indicates that while DBS significantly improves motor function in patients with PD, it does not induce widespread cognitive decline or worsening of mood symptoms. However, within the assessed cognitive domains, it may exert minor and specific negative effects on verbal fluency and complex executive control functions. This pattern is consistent with the known involvement of prefrontal-striatal circuits in these cognitive functions. Clinical decision-making requires individualized trade-offs between motor improvement and potential cognitive risks, accompanied by systematic preoperative assessment and postoperative monitoring. Future research should focus on optimizing DBS therapy through more precise target selection, parameter adjustment, and multimodal mechanistic investigations, ultimately enabling individualized and comprehensive management of both motor and non-motor symptoms in PD.

## Data Availability

The original contributions presented in the study are included in the article/Supplementary material, further inquiries can be directed to the corresponding authors.
