# Supplementary material for: Neuropsychological outcomes comparing deep brain stimulation and best medical treatment for Parkinson’s disease: a systematic review and meta-analysis
Source: Front Psychol. 2026 Jun 18;17:1865587. doi: 10.3389/fpsyg.2026.1865587 (PMC13322950; doi:10.3389/fpsyg.2026.1865587)
Supplement: Supplementary file 2 [file Data_Sheet_1.PDF]

search strategy

1.PubMed:

(("Non-Motor Symptoms"[Title/Abstract] OR "Nonmotor Symptoms"[Title/Abstract] OR "NMS"[Title/Abstract] OR "Cognition"[MeSH Terms] OR "Executive Function"[MeSH Terms] OR "Depression"[MeSH Terms] OR "Anxiety"[MeSH Terms] OR "cogniti*"[Title/Abstract] OR "Executive Function"[Title/Abstract] OR "verbal fluency"[Title/Abstract] OR "Depression"[MeSH Terms] OR "depress*"[Title/Abstract] OR "Anxiety"[MeSH Terms] OR "Anxiety"[Title/Abstract] OR "Dementia"[MeSH Terms] OR "Dementia"[Title/Abstract] OR "Memory"[MeSH Terms] OR "Memory"[Title/Abstract]) AND ("Parkinson Disease"[MeSH Terms] OR "Parkinsonian Disorders"[MeSH Terms] OR "parkinson*"[Title/Abstract] OR "Paralysis Agitans"[Title/Abstract]) AND ("Deep Brain Stimulation"[MeSH Terms] OR "DBS"[Title/Abstract] OR "deep brain stimulat*"[Title/Abstract] OR "neurostimulation"[Title/Abstract] OR "brain stimulat*"[Title/Abstract])) AND (randomizedcontrolledtrial[Filter])

2.Embase

Session Results

....

No.	Query Results	Results	Date
#5.	#1 AND #2 AND #3 AND [randomized controlled trial]/lim	420	24 Dec 2025
#4.	#1 AND #2 AND #3	9,028	24 Dec 2025
#3.	'cognition'/exp OR 'executive function'/exp OR 'depression'/exp OR 'anxiety'/exp OR 'verbal fluency'/exp OR 'dementia'/exp OR 'memory'/exp OR cogniti*:ab,ti OR depress*:ab,ti OR 'nonmotor symptoms':ab,ti	5,279,900	24 Dec 2025
#2.	'brain depth stimulation'/exp OR dbs:ab,ti OR 'deep brain stimulat*':ab,ti OR neurostimulation:ab,ti OR 'brain stimulat*':ab,ti	87,139	24 Dec 2025
#1.	'parkinson disease'/exp OR 'parkinsonian disorders':ab,ti OR parkinson*:ab,ti OR 'paralysis agitans':ab,ti	292,311	24 Dec 2025

3.Web of Science

((TI=("Parkinson Disease" OR "Parkinsonian Disorders" OR Parkinson* OR "Paralysis Agitans")) AND TI=("Deep Brain Stimulation" OR DBS OR "deep brain stimulat*" OR neurostimulation OR "brain stimulat*")) AND TI=("Non-Motor Symptoms" OR "Nonmotor Symptoms" OR "NMS" OR Cognition OR "Executive Function" OR Depression OR Anxiety OR cogniti* OR "verbal fluency" OR depress* OR Dementia OR memory)

4.Cochrane Library

#1 MeSH descriptor: [Parkinson Disease] explode all trees 6445

#2 ("Parkinsonian Disorders" OR Parkinson* OR "Paralysis Agitans"):ti,ab,kw 15554

#3 MeSH descriptor: [Deep Brain Stimulation] explode all trees 573

#4 (DBS OR neurostimulation):ti,ab,kw 2997

#5 ("nonmotor symptoms"):ti,ab,kw 889

#6 (Cognition OR "Executive Function" OR Depression OR Anxiety OR "verbal fluency" OR memory):ti,ab,kw 214104

#7 #1 OR #2 15554

#8 #3 OR #4 3154

#9 #5 OR #6 214563

#10 #7 AND #8 AND #9 269
